# A surrogate model for capturing wave power farm dynamics using spatial–temporal attention

**DOI:** 10.1038/s41598-026-64813-0

**Published:** 2026-08-03

**Authors:** Charitini Stavropoulou, Nicolás Faedo, Malin Göteman

**Affiliations:** 1 Department of Electrical Engineering, Uppsala University, Uppsala, Sweden; 2 Department of Mechanical and Aerospace Engineering, Politecnico di Torino, Turin, Italy; 3 Centre of Natural Hazards and Disaster Science (CNDS), Uppsala University, Uppsala, Sweden

**Keywords:** Wave energy farm, Spatial–temporal surrogate model, Transformer encoder, Point-absorber, Multi-device motion, Engineering, Mathematics and computing, Ocean sciences, Physics

## Abstract

Wave energy converters deployed in farms can experience intense hydrodynamic interactions due to the scattered and radiated waves on the free surface, making farm modeling challenging in realistic sea states. This study introduces a spatial–temporal surrogate model based on a transformer encoder architecture to predict the motion of multiple interacting wave energy converters in various sea states. The framework leverages experimental data from the SWELL dataset, predicting array responses in a previously unseen layout, i.e., a farm configuration, not available during the model’s training phase. The model embeds incident wave time series together with device coordinates into a unified spatial–temporal representation. Self-attention then jointly captures the temporal evolution of motion dynamics and inter-device spatial dependencies. Across three irregular sea states, the model predicts device responses with high accuracy, showing close agreement with experimental measurements. These findings provide an initial proof-of-concept, highlighting the potential of an attention-based spatial–temporal surrogate model as a building block for predicting the dynamics of several interacting wave energy converters in previously unseen array configurations.

## Introduction

Marine renewable energy has significant potential, not only due to the vast amount of available power globally, but also because of the high power density, estimated to be more than twenty times that of wind^1^. Despite extensive research, marine renewable energy, and wave energy in particular, is at an early stage of development, remaining one of the least tapped renewable energy resources. The limited maturity and the large diversity of wave energy technologies result in a wide range of levelized cost of energy (LCoE), creating an economic uncertainty that constrains large-scale investments and commercial deployment^2^. To create economically feasible projects, multiple wave energy converters (WECs) are expected to be deployed in arrays, creating wave power farms. This is particularly true for point-absorbers, which are among the most studied WEC concepts^3^. In essence, wave farms enable shared infrastructure, lower installation and maintenance costs per device, and smoother power output, thereby contributing to a reduced LCoE and enhanced grid integration.

When operating in farms, WECs experience intense hydrodynamic interactions due to the scattered and radiated waves at the free surface, making farm modeling challenging in realistic sea states. An understanding of the hydrodynamic interactions is essential for determining optimal array layout, accurate power estimation, and overall assessment of farm performance, key elements that govern the technological maturity and commercial feasibility of wave energy systems. High-fidelity numerical approaches, particularly computational fluid dynamics (CFD), have been employed to capture the wave-structure interaction, including viscous effects, turbulence, and wave-breaking phenomena. By discretising and numerically solving the Navier–Stokes equations, CFD models can resolve the complex flow patterns around individual devices, providing valuable insight into near-field dynamics. However, the high computational cost restricts CFD applications to studies of single WECs, making it impractical for simulating wave farms^4,5^. To recover computational efficiency, linear potential flow theory is typically employed^6^. Under the assumptions of a non-viscous, irrotational, and incompressible fluid, the governing equation for the fluid velocity potential reduces to the Laplace equation. By further assuming non-steep waves and small body motions, the boundary conditions at the free surface are linearized, and the total fluid velocity potential can be expressed as the superposition of incident, scattered, and radiated waves. To compute the potential and obtain the hydrodynamic coefficients, standard boundary element method or semi-analytical representations, e.g., the multiple-scattering approach^7^ can be used. The assumption of linear hydrodynamics leads to the time-domain formulation proposed by Cummins^8^, where the equations of motion for an array of interacting WECs subject to wave and other external forces reduce to a system of Volterra integro-differential equations of the convolution type, involving impulse response functions that describe wave excitation and radiation effects^9,10^. Assumptions associated with linear potential flow theory generally hold well for WEC operation in moderate sea states. However, significant discrepancies may arise in steep or energetic wave conditions, as the idealized approximations of the fluid flow can lead to overly simplified predictions, capturing only the general response trends while failing to reproduce the underlying dynamics accurately.

Recently, due to the increased data availability, machine learning approaches have gained significant attention in wave energy, where several studies explore data-driven models for wave forecasting, WEC behaviour, and farm-level performance. In^11^, a pyramid neural network is introduced using wind measurements from a reference point to accurately predict the significant wave height across nearshore and offshore wind and wave farms in the North Sea. In^12^, a time-delay neural network is proposed to estimate the instantaneous sea surface elevation across an open-field sea area for potential wave farm deployment using a single measurement point, demonstrating accurate wave reconstruction for short-term prediction purposes. Regarding wave energy technologies, in^13^ a multi-fidelity surrogate approach is introduced, combining low-fidelity numerical simulations based on linear potential flow theory with high-fidelity wave tank measurements to predict the heave motion of multiple interacting point-absorber WECs. Using a recurrent neural network to learn the mapping between the simplified and experimental responses, the model accurately reconstructs the real-time motion of the devices under irregular sea states, retaining low computational cost. Moreover, in^14^, a framework for wave farm power prediction is introduced, leveraging the spatial arrangement of WECs together with absorbed power measurements.

In many surrogate modeling approaches, incorporating multiple sources of information can significantly enhance the predictive performance. This is particularly relevant for signals collected from distributed sensors, where both spatial and temporal dependencies exist. When the arrangement of sensors is known, this information can be encoded through certain representations that capture spatial relationships. The recorded signals represent time series data that reflect the underlying system dynamics over time and, combined with the spatial information, form a spatial-temporal dataset. Spatial-temporal data structures naturally arise in wave energy applications, where the response of each WEC is influenced by the hydrodynamic interactions with neighbouring devices. Consequently, WECs located in proximity exhibit correlated motion patterns due to the scattered and radiated wave fields propagating throughout the farm. Thus, a WEC array reflects a distributed sensor network, where each device provides dynamic information that can be used by spatial-temporal learning frameworks.

Spatial-temporal surrogate methods seek to learn the relationship between multiple time-dependent variables. Within the renewable energy community, these modeling approaches have become increasingly popular. In^15^, a neural network architecture for wind farm power forecasting is introduced, combining a gated dilated-inception temporal module with a graph neural network to learn temporal dynamics and spatial interactions between turbines, including blockage effects. In^16^, a spatial-temporal model is introduced for multi-site offshore wind speed forecasting. Spatial dependencies between different measurement locations are captured through graph convolution, while temporal modeling is performed using a long-short-term memory neural network. The proposed framework achieves competitive performance on wind speed prediction across 120 offshore measurement sites in the China Sea. A similar approach has been explored in wave forecasting applications. In^17^, a spatial-temporal deep learning model is developed for short-term wave forecasting, where graph convolutions capture the spatial correlations among multiple wave measurement locations and a recurrent unit models the temporal evolution of the wave field.

Building on these developments, transformer architectures^18^ provide a natural framework for modeling WEC arrays, where the response of each device may be influenced by the dynamics of all other devices in the system. Their ability to capture long-range dependencies across time and space provides a global representation of the underlying system dynamics. In this setting, each transformer input represents the value of a variable at a specific location and time, enabling the model to relate measurements both across time and space. Through the self-attention mechanism, which evaluates pairwise correlations between inputs, transformers can dynamically weigh these relationships, capturing temporal patterns while learning interactions between spatially distributed sensors.

In^19^, a space–time transformer named EarthFormer is introduced for earth system forecasting. To address the increased computational cost of applying self-attention across large spatial-temporal grids, the model employs an attention mechanism, in which the input tensor is partitioned into small space–time blocks, called ’cuboids’, allowing attention to be computed efficiently. This design enables EarthFormer to achieve state-of-the-art performance on several climate forecasting benchmarks. Further, in^20^, EarthFormer is adapted to wave prediction in the northwestern Pacific Ocean, where sequences of wind field measurements from ERA5 are used to forecast wave characteristics, namely the significant wave height, mean wave period, and wave direction. The model successfully captures the evolution of waves over large oceanic regions and outperforms baseline deep learning methods. A complementary direction is explored in^21^, where a transformer is integrated into a diffusion framework for probabilistic wind speed forecasting. The model takes wind speed time series from several measurement sites as input, and employs attention to learn both the temporal evolution and the spatial correlations across the sensor network.

In this study, a unified spatial–temporal surrogate model is developed based on a transformer encoder architecture for predicting the angular motion of multiple interacting WECs. The model is trained on experimental data from the SWELL dataset^22^, using incident wave measurements and device positions, focusing on three irregular sea states. The experiments employ a 1:20 scale Wavestar point-absorber operating in angular pitch motion about a hinge axis. By learning the relationship between the incoming waves, the spatial arrangement of the devices, and their resulting motion, the surrogate model captures both the temporal evolution of the WEC dynamics as well as the spatial dependencies among the devices in each studied layout. Its ability to assess the motion predictions is evaluated on an *unseen* configuration, i.e., a new layout not available during the network’s training phase. While wave tank campaigns capture the full dynamics of multiple interacting WECs, they are limited to a small number of layouts and sea states due to time restrictions, facility availability, and operational cost. A surrogate model capable of predicting the dynamics of a previously unseen array configuration can facilitate the assessment of operating conditions beyond those directly represented in the training data, thereby extending the utility of experimental measurements. Under the constraints of the available dataset, the present results demonstrate that a purely data-driven surrogate can estimate the dynamics of several interacting WECs in an unseen incremental array configuration, providing an initial proof-of-concept and a foundation for future investigations involving more diverse wave farm layouts.

## Results

### Spatial-temporal surrogate model workflow

**Fig. 1:**
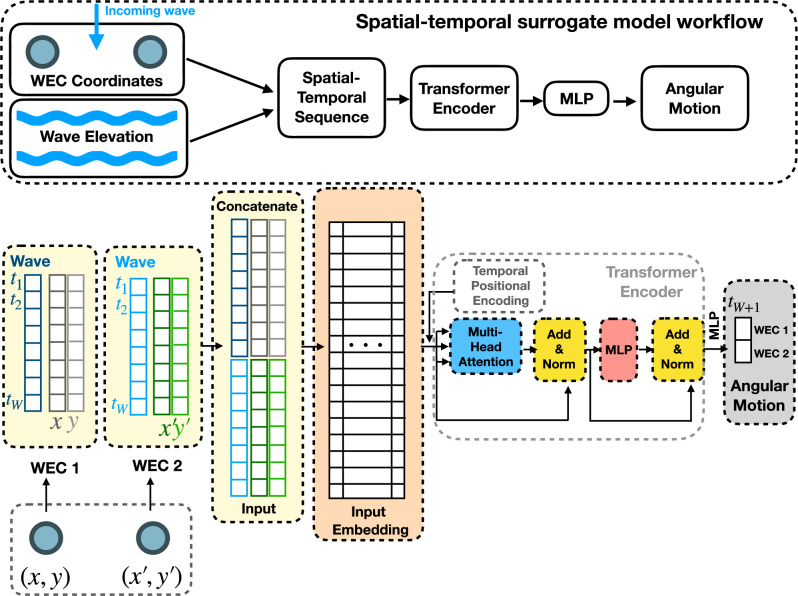
Spatial–temporal surrogate model workflow. For each layout, the incident wave elevation measurements are shaped into overlapping windows of length *W*, forming a sequence of short temporal segments that represent the wave field observed at the corresponding WEC locations. The wave elevation signals are combined with the spatial coordinates (*x*, *y*) of the corresponding WEC. For each layout, the resulting sequences for all the WECs are concatenated along the temporal dimension to form the input. Before being processed by the transformer encoder, the input is embedded into a latent dimension and augmented with temporal positional encoding to retain information on time ordering. The encoder processes the input using multi-head self-attention, residual connections, layer normalization, and a multilayer perceptron (MLP). A final MLP acting as an output head produces the angular displacement of all the WECs in the studied layout at $t_{W+1}$. The procedure is repeated across all temporal windows to predict the full angular motion time series for all the interacting WECs in each layout. One layout is illustrated here for simplicity; however, this framework can accommodate any number of training and testing layouts.

The workflow of the spatial-temporal model is shown in Fig. 1. For each farm layout, the model receives wave signals, recorded during empty tank tests, where probes are positioned at the locations corresponding to the WECs. The time series of wave elevation are handled using a sliding-window approach of fixed temporal length *W*, generating a collection of overlapping sequences. The resulting wave signals are combined with the location coordinates of the corresponding devices (*x*, *y*) to incorporate spatial context into the model input. If *N* is the number of WECs in each layout and *W* is the temporal sequence length, the multivariate input can be written as a tensor in $\mathbb {R}^{W\times N \times F}$ where each feature vector *F* contains the wave elevation at a given time step and the coordinates of the associated device. The multivariate input is flattened into a long sequence in $\mathbb {R}^{WN\times F}$ where each input token isolates the information associated with the incident wave at a single WEC at a specific time step. Therefore, for each layout, the input signals from all the WECs are concatenated along the temporal dimension, yielding one matrix that represents the temporal evolution of the incoming wave field and the spatial information of the layout. The resulting input sequence is embedded into a latent space that defines the internal feature representation processed by the transformer. A temporal positional encoding is added to preserve the temporal ordering of the samples within each sequence. The encoded input is passed through the self-attention mechanism, enabling the model to identify correlations between devices over time. The model outputs the angular displacement of all the interacting WECs within each studied layout.

**Fig. 2:**
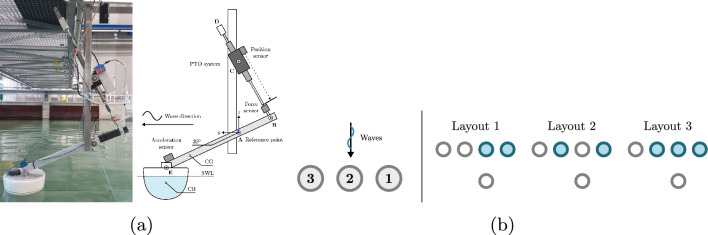
Wavestar prototype and studied layouts. In panel (**a**), the scaled Wavestar prototype used in the experiments is shown; the illustration is taken from^22^. In panel (**b**), a schematic representation of the WEC array layouts is shown. The coloured circles represent the actual positions of the WECs in each layout.

### Prediction of wave farm dynamics

**Fig. 3:**
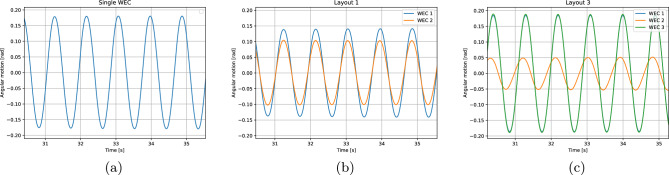
Illustrative comparison of angular motion for increasing array complexity under near-resonant conditions. The response of a single WEC (**a**), two interacting WECs in layout 1 (**b**), and three interacting WECs in layout 3 (**c**) is shown. While the single and two-device configurations exhibit relatively consistent response patterns, the three-device configuration introduces a distinct motion behavior, particularly for the intermediate WEC, which is influenced by both neighboring devices. This reflects the transition from pairwise to multi-body hydrodynamic interactions.

The goal of the spatial–temporal surrogate approach is to predict the angular displacement of multiple interacting WECs arranged in arrays and to estimate these dynamics for a previously unseen layout, i.e., a configuration not available during the network’s training phase. More specifically, the network is trained on two row-like configurations, layouts 1 and 2, see Fig. 2, each containing two WECs and subjected to three irregular sea states, ISS1, ISS2, and ISS3 generated stochastically within the wave tank in terms of a JONSWAP spectrum, see Table 1. Note that, as extensively discussed in SWELL^22^, these three sea states effectively excite a wide range of operating conditions, representative of the intended operation site. In both training layouts, the two interacting devices are positioned along a line perpendicular to the incoming wave direction, while each layout has different inter-device spacings, introducing distinct levels of hydrodynamic interaction. The evaluation of the surrogate model is performed on layout 3, containing three devices arranged in a row-like configuration, thus introducing an incremental set-up. The validation scenario is designed to assess the model’s predictive ability, as the three-device configuration introduces interaction patterns not explicitly present in the training data. In layout 3, the addition of a third intermediate device modifies the wave field, creating coupled dynamics that require the model to infer motion beyond previously seen pairwise interactions. This configuration results in a multi-body interaction regime, in which the central device is simultaneously influenced by both neighboring WECs under hydrodynamic conditions that lead to coupled responses that cannot be decomposed into independent pairwise contributions. To further illustrate this transition, Fig. 3 presents a simplified comparison of the angular motion for a single device, a two-device configuration (layout 1), and a three-device configuration (layout 3) under near-resonant conditions. While the response of the two-device layout reflects pairwise interactions between neighboring WECs, the three-device configuration exhibits a distinct behavior, particularly for the intermediate unit. The central WEC displays a modified response pattern arising from the simultaneous influence of both adjacent devices, reflecting coupled dynamics that extend beyond pairwise interactions.

**Table 1:** Conditions ISS1–ISS3. Each wave follows a JONSWAP spectrum. $T_p$ is the peak period and $H_s$ is the significant wave height.

Sea state	T$_p$ [s]	H$_s$ [m]	Length [s]
ISS1	1.412	0.063	300
ISS2	1.836	0.104	300
ISS3	0.988	0.0208	300

**Fig. 4:**
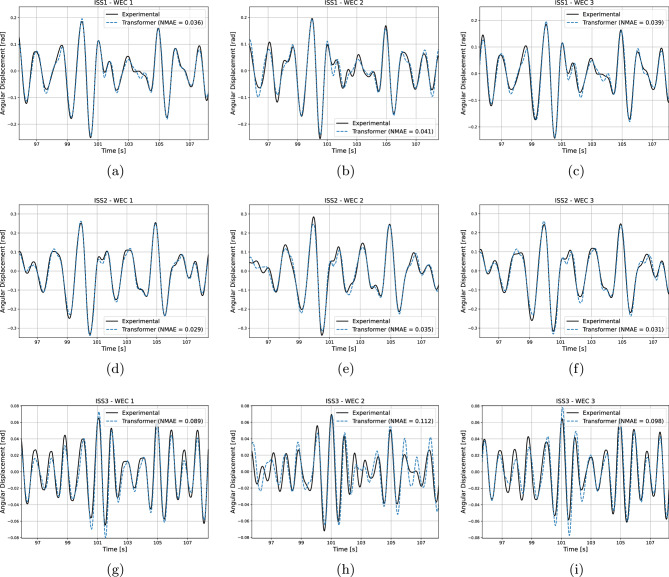
Comparison of time series for layout 3. Time-series predictions of angular displacement for layout 3 under irregular sea states ISS1–ISS3. Panels (**a**–**c**) correspond to ISS1, (**d**–**f**) to ISS2, and (**g**–**i**) to ISS3. For each sea state, the three WECs are shown side by side. The predicted angular motion (dashed line) is compared against experimental measurements (solid line), demonstrating the ability of the surrogate model to reproduce the motion of multiple interacting WECs. The normalized mean absolute error (NMAE) between predicted and experimental angular displacement is reported in each panel.

The network is used to predict the angular displacement of all the WECs in layout 3 under ISS1–ISS3, thereby evaluating its ability to predict the dynamics of a previously unseen array configuration under different wave conditions. Fig. 4 shows the time series predictions of angular displacement for all WECs in layout 3 under conditions ISS1–ISS3. For every sea state, the predicted response of each WEC is plotted against the corresponding experimental measurements, showing good agreement in both amplitude and phase. The time intervals shown in Fig. 4 correspond to segments of the experimental measurements that include the highest motion peaks, providing a representative view of the system dynamics. To ensure consistency across devices, the same interval is used for all WECs within each sea state. In ISS3, this interval is selected around the dominant motion peak of the intermediate WEC, within the same segment; the outer devices exhibit large-amplitude responses, allowing the comparison to remain aligned with the rest of the cases. To quantify the resulting differences in device-specific responses, a cross-correlation analysis is performed between all WEC pairs in layout 3, see Table 3. The results show that, while the outer devices remain highly correlated, the middle WEC exhibits reduced correlation with both neighbors, with the discrepancy becoming most pronounced in the near-resonant sea state ISS3, where hydrodynamic interactions are stronger. The predicted time series are evaluated against the experimental angular displacement using the normalized mean absolute error (NMAE), see equation (14). The NMAE provides a direct measure of the overall discrepancy between the predicted and experimental responses, the latter serving as the ground truth. In this study, the NMAE is calculated for each WEC in layout 3 in all wave scenarios, as summarized in Fig. 4. To account for differences in motion amplitude across devices and sea states, the mean absolute error is normalized by the maximum absolute value of the corresponding experimental angular displacement for each WEC.

**Table 2:** Hyperparameters defining the surrogate model structure and training configuration.

Parameter	Symbol	Value
Window size	*W*	500
Batch size	B	4
Encoder layers	*L*	1
Attention heads	*H*	4
Latent dimension	$d_{\text {model}}$	64
Feed-forward dimension	$d_{\text {ff}}$	128
Dropout rate	–	0.2
Learning rate	–	0.001
Optimizer	–	Adam

**Table 3:** Cross-correlation coefficients between measured angular responses of WEC pairs in layout 3 for each sea state, see Eqs (12)–(13). The results indicate consistently high similarity between the outer devices (WEC1–WEC3), while the middle device (WEC2) exhibits reduced correlation with both outer WECs, particularly close to the resonant sea state ISS3.

Sea state	WEC1–WEC3	WEC1–WEC2	WEC3–WEC2
ISS1	0.995	0.888	0.891
ISS2	0.993	0.937	0.936
ISS3	0.986	0.698	0.689

**Fig. 5:**
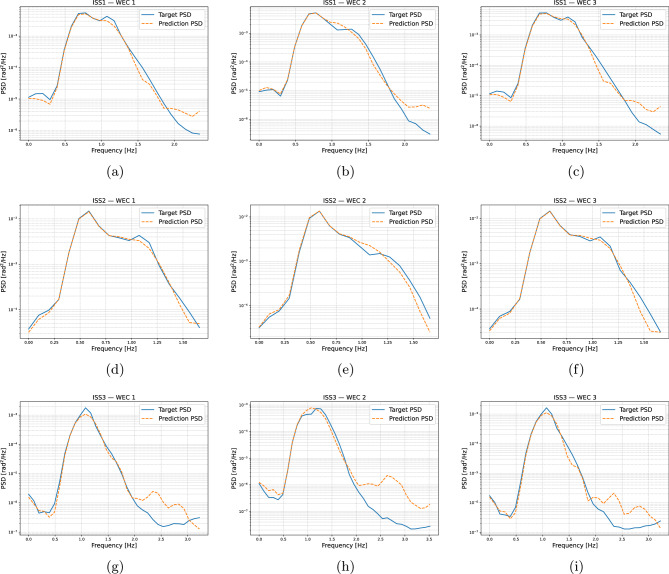
Comparison of PSD for layout 3. PSD assessment between predicted and experimental angular displacement for all WECs in layout 3 under ISS1–ISS3. Panels (**a**–**c**) correspond to ISS1, (**d**–**f**) to ISS2, and (**g**–**i**) to ISS3. Each panel compares the PSD of the predicted motion (dashed line) with that of the experimental measurement (solid line), showing that the model accurately reproduces the dominant frequencies and overall energy distribution for each WEC.

The agreement between the predicted and experimental angular displacements is further examined through a power spectral density (PSD) analysis, see equation (15), shown in Fig. 5. For each WEC and sea state, the predicted PSD is compared with the corresponding experimental spectrum to assess the model’s ability to reproduce the dominant frequencies of motion. Across all three irregular waves, the predicted spectra follow the experimental distributions, accurately capturing the peak frequency and the overall energy content. Deviations appear primarily at higher frequencies, which correspond to very short wave periods that are less relevant under typical operating conditions and are more sensitive to noise and temporal resolution.

## Discussion

The results demonstrate that the proposed transformer encoder surrogate model can predict the angular motion of several interacting WECs for a previously unseen farm layout when provided only with incident wave elevation at the device locations and their spatial coordinates. By treating each WEC–time pair as an individual token within a single sequence, the model learns a joint spatial–temporal representation of the farm dynamics. This unified treatment allows the self-attention mechanism to identify correlations along the incoming wave history at a given WEC and across devices within each layout. In the scenario considered here, the model is trained on two row-like layouts, each containing two WECs with different inter-device spacings, and then evaluated on a three-device row layout. The ability of the surrogate to predict the motion across all WECs in the new incremental set-up, including the altered responses induced by the intermediate unit, demonstrates that the model has learned a non-trivial mapping from incident wave input and spatial arrangement to device motion.

Additionally, the evaluation across three irregular wave conditions demonstrates that the surrogate maintains good predictive performance under varying operating conditions. More specifically, ISS1 and ISS2, which are narrow-banded and excite the devices over a relatively confined frequency range, are predicted with consistently low error. In contrast, ISS3 has a substantially broader spectrum that includes near-resonant, low, and higher-frequency components, leading to excitation near the devices’ natural period. Near-resonant excitation amplifies the hydrodynamic coupling between devices, making their motion more sensitive to discrepancies in the learned dynamics, accounting for the higher errors obtained for ISS3. For all sea states, slightly larger errors occur at the intermediate device in the evaluation layout. This behavior is expected, as the middle WEC experiences hydrodynamic coupling, influenced by the scattered and radiated waves from both neighboring units. We note that such interaction patterns do not explicitly appear in the training layouts, which contain only pairwise configurations. Consequently, the model infers additional coupling solely from the available data inputs, i.e., the incident wave time series and device coordinates, in the absence of any direct representation of the governing hydrodynamic equations. The slightly higher error at the intermediate WEC reflects the inherent difficulty of predicting the farm dynamics in a new configuration whose interaction structure is only partially captured by the training data, rather than a limitation of the modeling framework.

The proposed surrogate model illustrates the potential of a purely data-driven approach to support efficient evaluation of wave farm configurations. Once trained, the transformer can predict the motion of an entire array at low computational cost, enabling the screening of candidate layouts. This functionality may be valuable during the preliminary stages of wave farm development, where candidate layouts are often considered and detailed numerical or experimental investigations may be impractical. Although more complex farm geometries may benefit from additional physical constraints, the present study demonstrates that attention-based spatial–temporal surrogates provide an effective pathway toward efficient, data-driven analysis of array dynamics. The current testing is performed using the row-like configurations available within the SWELL dataset, where the model is trained on two-device layouts and evaluated on a previously unseen three-device configuration. While broader assessment across more diverse array geometries requires additional training layouts and experimental observations, the present results demonstrate the ability of the proposed framework to transfer information learned from simpler configurations to a new array arrangement not encountered during training. As such, this work serves as an initial proof-of-concept for the use of attention-based spatial–temporal surrogates in predicting the dynamics of previously unseen WEC array configurations.

## Methods

### Experimental dataset description

This study uses the SWELL dataset (Standardised Wave Energy Converter Array Learning Library)^22^, a high-fidelity experimental benchmark developed at the Ocean and Coastal Engineering Laboratory at Aalborg University. The dataset comprises wave tank experiments on scaled WEC arrays performed across multiple sea states. The experiments employ a 1:20 scale prototype of the Wavestar point-absorber WEC, consisting of a buoy hinged to a fixed support frame above the still-water level, see Fig. 2. A direct-drive linear motor, mounted at the hinge, serves as the PTO system. The experimental campaign includes four complementary test types: empty tank wave measurements, free uncontrolled motion of the WECs, controlled motion, and locked-device tests for excitation force characterization. In this study, the data from the free motion tests are used, where the devices move in a single degree of freedom about the hinge axis. The responses of the WECs are recorded using high-resolution sensors synchronised at 200 Hz. Measurement uncertainty, repeatability, and accuracy are documented in detail in the source dataset, where controlled testing conditions and consistent procedures are reported to minimise measurement variability and its propagation. From these measurements, the angular displacement of each device is obtained and used as the target variable for model development.

The campaign considers several WEC prototypes, which can be mounted or removed from the basin to form different layout configurations. In Fig. 2 the layouts used in this study are shown. Layouts 1 and 2 each consist of two devices arranged in a line perpendicular to the incoming wave direction, with different spacing between the WECs to vary the level of hydrodynamic interaction between neighbouring units. Layout 3 consists of three devices arranged in a row. The chosen set of layouts enables a systematic investigation of how hydrodynamic interactions vary with device spacing and with an incremental number of operating units. Three conditions, ISS1, ISS2, and ISS3, are used to investigate the responses of the studied layouts. All sea states are generated in the wave basin and follow the JONSWAP spectrum. Each wave is defined by a significant wave height H$_s$ and a peak period T$_p$, see Table 1. ISS1 and ISS2 correspond to narrow-banded conditions, while ISS3 represents a broader-banded sea state with energy distributed across low-, resonance-, and high-frequency components. Measurements of the free-surface elevation are obtained using wave probes at selected locations within the basin. These measurements provide localized characterization of the wave conditions at the probe positions, rather than a full reconstruction of the spatial–temporal wave field across the entire farm domain. During the empty tank tests, the probes positioned at the locations corresponding to the WECs in the studied layouts record the incident wave elevation, which is used as the input reference.

### Transformer encoder model

Let

$$\textbf{X} \in \mathbb {R}^{N W \times F},$$

denote the sequence of input tokens, where *N* is the number of WECs per layout, *W* is the temporal window length, and *F* is the number of features per token. Each token is associated with a particular WEC-time pair,

$$\textbf{x}_t \in \mathbb {R}^{F},$$

containing the wave elevation *η* and the spatial coordinates (*x*, *y*) of the corresponding WEC at time *t*. The transformer processes all tokens in parallel, allowing the model to learn both temporal and inter-device spatial correlations.

#### Input embedding and positional encoding

Each token $\textbf{x}_t = (\eta , x, y)$ is embedded in the latent dimension $d_{\textrm{model}}$ by combining an embedding of the wave elevation with an embedding of the WEC coordinates. The scalar wave elevation *η* is embedded using a linear projection,

$$\textbf{z}^{(\eta )} = \eta \, W_{\eta } + b_{\eta }, \qquad W_{\eta } \in \mathbb {R}^{1 \times d_{\textrm{model}}},\; b_{\eta } \in \mathbb {R}^{1 \times d_{\textrm{model}}}.$$

This operation is applied row-wise to the sequence $\eta \in \mathbb {R}^{NW}$. The spatial coordinates (*x*, *y*) are embedded using a two-layer MLP,

$$\textbf{z}^{(c)} = \sigma \!\left( [x,y]\,W_{1} + b_{1}\right) W_{2} + b_{2},$$

where

$$W_{1} \in \mathbb {R}^{2 \times d_{\textrm{model}}}, \qquad b_{1} \in \mathbb {R}^{1 \times d_{\textrm{model}}},$$

$$W_{2} \in \mathbb {R}^{d_{\textrm{model}} \times d_{\textrm{model}}}, \qquad b_{2} \in \mathbb {R}^{1 \times d_{\textrm{model}}}.$$

Here $\sigma (\cdot )$ denotes the ReLU nonlinear activation function. Similarly, the MLP is applied row-wise. The full embedding of each token is obtained by summation,

$$\textbf{Z} = \textbf{z}^{(\eta )} + \textbf{z}^{(c)}, \qquad \textbf{Z} \in \mathbb {R}^{NW \times d_{\textrm{model}}}.$$

Since the self-attention mechanism is permutation invariant, a sinusoidal positional encoding $\textbf{P} \in \mathbb {R}^{NW \times d_{\textrm{model}}}$ is applied per WEC, to introduce temporal ordering in the sequence. This encoding provides each token with a unique representation, allowing the model to capture temporal dependencies within the input sequence,

1 $$\begin{aligned} \textbf{H}_0 = \textbf{Z} + \textbf{P}. \end{aligned}$$

More specifically, each token $\textbf{z}_t$ at position *t* is augmented with an encoding defined as

2 $$\begin{aligned} P_{t,2i} = \sin \!\left( \frac{t}{10000^{2i/d_\text {model}}}\right) , \qquad P_{t,2i+1} = \cos \!\left( \frac{t}{10000^{2i/d_\text {model}}}\right) , \end{aligned}$$

for $$i = 0, \dots , d_\text {model}/2 - 1$$.

#### Self-attention mechanism

The core operation of the transformer is the self-attention mechanism, which computes a weighted combination of all tokens based on their pairwise similarity. For a given layer input $\textbf{H}_{l-1}\in \mathbb {R}^{NW\times d_\text {model}}$, three linear projections generate the query, key, and value matrices,

3 $$\begin{aligned} \textbf{Q} = \textbf{H}_{l-1}\textbf{W}^Q,\qquad \textbf{K} = \textbf{H}_{l-1}\textbf{W}^K,\qquad \textbf{V} = \textbf{H}_{l-1}\textbf{W}^V, \end{aligned}$$

where $\textbf{W}^Q,\textbf{W}^K,\textbf{W}^V \in \mathbb {R}^{d_\text {model}\times d_k}$ are learnable weight matrices. The self-attention weights are obtained via the scaled dot-product between the queries and keys as,

4 $$\begin{aligned} \textbf{A} = \textrm{softmax}\!\left( \frac{\textbf{Q}\textbf{K}^\top }{\sqrt{d_k}}\right) . \end{aligned}$$

Each row of $\textbf{A}\in \mathbb {R}^{NW\times NW}$ is normalized by the softmax function, ensuring that all attention weights are non-negative and sum to one. The output of the self-attention operation is then,

5 $$\begin{aligned} \textbf{S} = \textbf{A}\textbf{V}. \end{aligned}$$

This mechanism updates each token as a weighted combination of all others, where the weights are dynamically learned from the data and quantify the strength of their pairwise relevance.

#### Multi-head self-attention

To capture several types of dependencies in the data, the transformer employs multiple attention heads. For head *h=1,… ,H*, the above operation is repeated with projections $\textbf{W}_h^Q, \textbf{W}_h^K, \textbf{W}_h^V$, and their outputs are concatenated and linearly transformed,

6 $$\begin{aligned} \textrm{T}(\textbf{H}_{l-1}) = \textrm{Concat}(\textbf{S}_1, \dots , \textbf{S}_H)\textbf{W}^O, \qquad \textbf{W}^O \in \mathbb {R}^{H d_k \times d_{\text {model}}}. \end{aligned}$$

The multi-head self-attention operation allows different heads to focus on various dependencies, improving the model’s ability to represent complex relationships in the data.

#### Transformer encoder layer structure

Each transformer encoder layer processes the input $\textbf{H}_{l-1}\in \mathbb {R}^{NW\times d_{\text {model}}}$ using four components, namely the multi-head self-attention, residual connections, layer normalization, and an MLP. For layer *l=1,… ,L*, the update equations are

7 $$\begin{aligned} \textbf{U}_l&= \textrm{LayerNorm}\!\left( \textbf{H}_{l-1} + \textrm{T}(\textbf{H}_{l-1}) \right) , \end{aligned}$$

8 $$\begin{aligned} \textbf{H}_l&= \textrm{LayerNorm}\!\left( \textbf{U}_l + \textrm{MLP}(\textbf{U}_l) \right) . \end{aligned}$$

The same MLP operation is applied across all tokens $\textbf{u}$ in $\textbf{U}_l$, and consists of two linear transformations and a ReLU nonlinear activation function $\sigma (\cdot )$.

9 $$\begin{aligned} \begin{aligned} \textrm{MLP}(\textbf{u})&= \sigma (\textbf{u}\textbf{W}_1 + \textbf{b}_1)\textbf{W}_2 + \textbf{b}_2, \\ \textbf{u}&\in \mathbb {R}^{1\times d_\text {model}}, \quad \textbf{W}_1 \in \mathbb {R}^{d_\text {model}\times d_\text {ff}}, \quad \textbf{b}_1 \in \mathbb {R}^{1\times d_\text {ff}}, \\ \textbf{W}_2&\in \mathbb {R}^{d_\text {ff}\times d_\text {model}}, \quad \textbf{b}_2 \in \mathbb {R}^{1\times d_\text {model}}. \end{aligned} \end{aligned}$$

The residual connections in Eqs. (7)–(8) ensure stable gradient propagation, while layer normalization maintains consistent feature scaling across all tokens.

#### Output projection.

The encoder output $\textbf{H}_L\in \mathbb {R}^{NW\times d_\text {model}}$ collects the latent features of all the interacting WECs across the given temporal window. For all WECs, the embedding at the last time step $\textbf{H}_L^{(\text {last})}\in \mathbb {R}^{N\times d_\text {model}}$ is extracted and mapped to the output dimension through a linear projection,

10 $$\begin{aligned} \widehat{\textbf{Y}} = \textbf{H}_L^{(\text {last})}\textbf{W}_\text {out} + \textbf{b}_\text {out}, \qquad \textbf{W}_\text {out}\in \mathbb {R}^{d_\text {model}\times 1}, \; \textbf{b}_\text {out}\in \mathbb {R}. \end{aligned}$$

Here $\widehat{\textbf{Y}}\in \mathbb {R}^{N\times 1}$ denotes the resulting predictions of the WECs’ angular displacement.

### Data normalization

All variables are normalized to ensure stable optimization and consistent scaling across features. The wave elevation time series *η* are standardized per probe as,

$$\eta ^{\textrm{norm}} = \frac{\eta - \mu _\eta }{\sigma _\eta },$$

where the mean $\mu _\eta$ and standard deviation $\sigma _\eta$ are computed from each measured wave. The angular motion time series are standardized per WEC as,

$$y^{\textrm{norm}} = \frac{y-\mu _m}{\sigma _m},$$

where $\mu _m$ and $\sigma _m$ are computed exclusively from the training layouts. The spatial coordinates (*x*, *y*) are standardized in the same manner.

### Model definition and training objective

The performance of the transformer depends on several key hyperparameters. These include the latent dimension of the transformer encoder $d_{\text {model}}$, the number of attention heads *H*, the feed-forward dimension $d_{\text {ff}}$, and the temporal window size *W*, which defines the number of consecutive time steps of wave history considered by the model to predict the angular displacement. A larger window size allows the model to capture longer dependencies in the data at the cost of increased computational demand and higher memory usage. The temporal window is advised to span at least one full period for each wave, ensuring that the model receives at least a complete cycle of wave excitation for every prediction step.

**Fig. 6:**
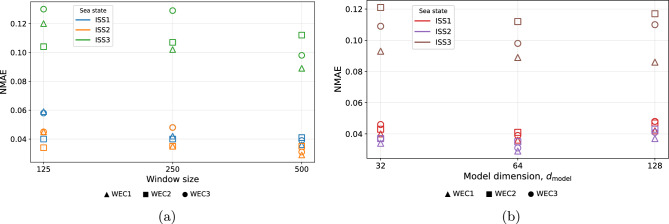
(**a**) Sensitivity analysis of the input window size. The NMAE (equation (14)) obtained for the three sea states (ISS1–ISS3), and the three WECs is shown for window sizes of 125, 250, and 500 time steps, while all remaining model hyperparameters are kept fixed at their baseline values. (**b**) Sensitivity analysis of the transformer latent dimension, $d_{\textrm{model}}$. The NMAE (equation (14)) obtained for the three sea states (ISS1–ISS3) and the three WECs is shown for $d_{\textrm{model}}=$32, 64, and 128 units, while all other hyperparameters are maintained at their baseline values.

The model hyperparameters listed in Table 2 are selected to balance computational efficiency and predictive accuracy with respect to the available dataset size. The model depth and latent dimensions are kept moderate to ensure sufficient expressiveness, the number of attention heads enables the capture of multiple spatial–temporal patterns, and the batch size reflects the memory constraints associated with long input sequences. A dropout rate of 0.2 is applied within the transformer encoder to regularize the network and reduce overfitting. To assess the robustness of the selected configuration, a sensitivity analysis is conducted by varying the temporal window size and the transformer latent dimension while maintaining all other hyperparameters at their baseline values. As shown in Fig. 6, increasing the window size generally improves prediction accuracy by providing additional temporal context, whereas increasing the model dimension beyond moderate values yields comparatively limited gains. The model is trained using the Adam optimization algorithm^23^, with an adaptive learning rate initialised to 0.001. The learning rate is reduced during training using a scheduler that decreases it by a factor of 0.5 if the validation loss does not improve for ten consecutive epochs. To prevent overfitting and unnecessary computations, an early stopping criterion is employed with a patience of thirty epochs. If there is no improvement in the validation loss for thirty consecutive epochs, the training is terminated, and the model corresponding to the minimum validation loss is retained. All experiments are performed on a high-performance computing node equipped with an NVIDIA T4 GPU (16 GiB VRAM) and dual Intel Xeon Gold 6130 CPUs (96 GiB RAM). The training process required approximately 15 hours. To assess the sensitivity of the model to stochastic training effects, the complete training procedure is repeated three independent times. The resulting NMAE values, equation (14), exhibited limited variability, with an average coefficient of variation of 4.9% and a maximum value of 11.2% across all WECs and sea states, indicating relatively stable predictive performance with respect to random initialization and optimization trajectories.

The model is trained on layouts 1 and 2, each containing two devices, using ISS1–ISS3. Its performance is evaluated on a new configuration, layout 3, including three devices, for each sea state. Training proceeds by minimizing a loss function that quantifies the discrepancy between the predicted and experimental angular displacement for each WEC. In this study, the mean squared error (MSE) is used as,

11 $$\begin{aligned} \text {MSE} = \frac{1}{sn}\sum _{i=1}^s\sum _{j=1}^n( y_{i,j}^{true}- y_{i,j}^{pred})^2. \end{aligned}$$

**Fig. 7:**
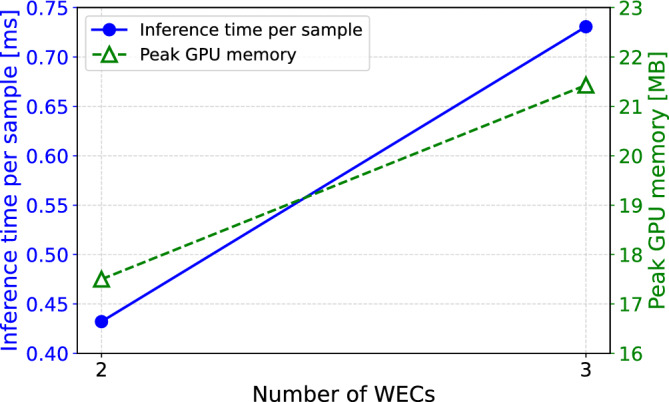
Inference time per sample and peak GPU memory usage as a function of the number of WECs. One sample corresponds to a single prediction time step, where the model predicts the angular displacement of all WECs in the array. Measurements are obtained using an NVIDIA Tesla T4 GPU.

To train the network, the dataset is partitioned into several batches, each batch consisting of *s* samples. Each sample corresponds to a prediction time step *i*. The training data is split into 80% training and 20% validation subsets. The split is performed along the temporal dimension before applying the sliding-window preprocessing. Training and validation windows are generated independently within their respective segments. Given approximately $6 \times 10^{4}$ time steps per case, this results in on the order of $2.8 \times 10^{5}$ training samples for all layouts and sea states. In equation (11), the discrepancy between the experimental angular motion $y^{true}$ and the predicted value $y^{pred}$ is computed for each sample, and the error is averaged over the batch. Since the network involves *n* outputs per sample, namely the number of WECs within each layout, the mean error across the outputs is taken. To provide an indication of the computational behavior, the inference time per sample and peak GPU memory usage are evaluated for the available two-WEC and three-WEC configurations. As shown in Fig. 7, increasing the array size from two to three WECs increased the inference time per sample from $4.32 \times 10^{-4}\,\textrm{s}$ to $7.31 \times 10^{-4}\,\textrm{s}$, while the peak GPU memory increased from $17.50\,\textrm{MB}$ to $21.43\,\textrm{MB}$, given the selected batch size. These results illustrate the computational characteristics of the proposed framework within the range of array sizes represented in the available experimental dataset.

### Evaluation metrics

To evaluate the similarity between the experimental angular response signals of different WECs, we employ the cross-correlation, which evaluates the degree of agreement between two time series defined as,

12 $$\begin{aligned} \rho _{xy}(k) = \frac{\sum _{i=1}^{T} \left( x_i-\mu _x\right) \left( y_{i-k}-\mu _y\right) }{T\ \sigma _x\ \sigma _y} \end{aligned}$$

13 $$\begin{aligned} \rho _{xy}^{\max } = \max _{k} \rho _{xy}(k). \end{aligned}$$

Here, $x_i$ and $y_i$ denote the angular displacement time series of two WECs, *i* is the discrete time index, and *T* is the total length of the signals. The quantities $\mu _x$ and $\mu _y$ represent the mean values of the respective signals, while $\sigma _x$ and $\sigma _y$ denote their standard deviations. The variable *k* corresponds to the discrete lag, in time steps, between the two signals. The coefficient $\rho _{xy}(k)$ measures the similarity at a given lag, and $\rho _{xy}^{\max }$ represents the maximum correlation over all considered lags. This formulation provides a single similarity measure for each pair of WEC responses while accounting for possible phase differences between signals.

To quantify the model’s accuracy, we compute the NMAE for each WEC using the formula

14 $$\begin{aligned} \text {NMAE} = \frac{\frac{1}{T} \sum _{i=1}^{T}|y^{true}_i - y^{pred}_i|}{\max {|\boldsymbol{y} ^{true}|}}, \end{aligned}$$

where *T* is the length of the evaluated time series, $y^{true}_i$ is the experimental angular displacement, $y^{pred}_i$ is the predicted value, and $\boldsymbol{y}^{true}$ denotes the full experimental time series for the corresponding WEC.

To assess the agreement between the predicted and experimental angular motion in the frequency domain, the PSD of each signal is evaluated. For a discrete-time signal $y(t_i)$ sampled at frequency $f_s$, the PSD is defined as

15 $$\begin{aligned} S_{y}(f) = \lim _{T \rightarrow \infty } \frac{1}{T} \left| \int _{0}^{T} y(t)\, e^{-\,\textrm{i} 2\pi f t}\, dt \right| ^{2}, \end{aligned}$$

which reveals the contribution of different frequencies to the overall motion and thus highlights the dominant oscillatory components governing the WEC response. In practice, the PSD is estimated using Welch’s method^24^, in which the signal is divided into overlapping segments, each segment is multiplied by the Hann window, transformed into the frequency domain, and the resulting spectra are averaged. This procedure reduces the variance of the spectral estimate while preserving the dominant dynamical features of the motion.

## Data Availability

The experimental data used in this study are part of the SWELL dataset^22^. Wave elevation and angular displacement measurements analysed in this work are available at the repository https://doi.org/10.17632/n34wcksmts.2.

## Code availability

The code used in this study is available from the corresponding author upon reasonable request.
